# miRNAs Regulate Cytokine Secretion Induced by Phosphorylated S100A8/A9 in Neutrophils

**DOI:** 10.3390/ijms20225699

**Published:** 2019-11-14

**Authors:** Nicolas Jung, Véronique Schenten, Jean-Luc Bueb, Fabrice Tolle, Sabrina Bréchard

**Affiliations:** Immune Cells and Inflammatory Diseases group, Life Sciences Research Unit, University of Luxembourg, L-4367 Belvaux, Luxembourgveronique.schenten@uni.lu (V.S.); jean-luc.bueb@uni.lu (J.-L.B.); fabrice.tolle@uni.lu (F.T.)

**Keywords:** miRNAs, cytokines, inflammation, S100A8/A9, neutrophils

## Abstract

The release of cytokines by neutrophils constitutes an essential process in the development of inflammation by recruiting and activating additional cells. Neutrophils are also able to secrete a complex of S100A8 and S100A9 proteins (S100A8/A9), which can amplify the general inflammatory state of the host and is involved in the pathogenesis of several chronic inflammatory diseases, such as rheumatoid arthritis (RA). S100A8/A9 have received renewed attention due to their susceptibility to several function-altering post-translational modifications. In that context, it has been recently demonstrated that only the phosphorylated form of S100A8/A9 (S100A8/A9-P) is able to induce the secretion of several cytokines in neutrophils. Here, we investigate the mechanism by which this post-translational modification of S100A8/A9 can regulate the extracellular activity of the protein complex and its impact on the inflammatory functions of neutrophils. We found that S100A8/A9-P are present in large amounts in the synovial fluids from RA patients, highlighting the importance of this form of S100A8/A9 complex in the inflammation process. Using miRNA-sequencing on S100A8/A9-P-stimulated differentiated HL-60 cells, we identified a dysregulation of miR-146a-5p and miR-155-5p expression through TRL4 signaling pathways. Our data reveal that overexpression of these miRNAs in neutrophil-like cells reduces S100A8/A9-P-mediated secretion of pro-inflammatory cytokines.

## 1. Introduction

The most evident phenomenon concerning neutrophils is the remarkable evolution of the researchers’ view of their role over the years. They have passed from the status of elementary cells dedicated to pathogen killing to that of flexible and adaptive cells coordinating the immune response. One of the critical elements to achieve such a determinant function is their ability to secrete a large spectrum of cytokines, which recruit additional neutrophils and immune cells leading ultimately to an amplification of the immune response [1,2,3]. 

Most cytokines originate from de novo synthesis, but preformed cytokines are also stored and thus rapidly released upon appropriate stimulation [4,5]. This capacity of neutrophils to secrete a specific cytokine profile depending on the underlining conditions [6] may determine their potential to influence the development of inflammation and trigger pathological outcomes.

Neutrophils can also contribute to the perpetuation of the inflammatory process through the secretion of S100A8 and S100A9, two EF-hand Ca^2+^-binding proteins, which have the ability to form a protein complex (S100A8/A9, also called calprotectin). These proteins are now qualified as damage-associated molecular pattern molecules (DAMPs) [7,8] due to their capacity to act as cytokine-like proteins and possibility to recruit other cells and amplify the overall inflammatory state. In this sense, S100A8/A9 have been described as being involved in the pathogenesis of chronic inflammatory diseases and more notably in rheumatoid arthritis (RA) [9,10,11]. Indeed, S100A8/A9 are markedly up-regulated in the synovial fluid or serum of RA patients and can be used as sera-based biomarkers to evaluate disease severity and progression [12,13]. 

The role of S100A8/A9 in disease development and progression is tightly related to their pro-inflammatory activities [14,15]. The latter are primarily associated with the active heterodimer form (S100A8/A9) [16], and recently, it has been established that the post-translational modification of S100A9 is a critical event to regulate the DAMP functions of S100A8/A9 [17]. In this regard, based on the use of DMSO-differentiated HL-60 cells (dHL-60), the phosphorylated form of S100A9 within the S100A8/A9 complex (S100A8/A9-P) has recently been shown to induce the expression and secretion of several cytokines. This event has been identified as mostly occurring through the activation of Toll-like receptor 4 (TLR4) signaling pathways, although the involvement of other receptors cannot be excluded. In fact, S100A8/A9-P could activate different types of receptors, which could allow the secretion of different cytokine profiles. In that respect, TNFα, CCL3, CCL4, IL-6 and CXCL8 release appear to be dependent on TLR4 signaling, whereas the release of CCL2 seems to be associated with a distinct downstream signaling pathway activated by a distinct receptor [17]. The release of such cytokines by neutrophils can participate in the dysregulation of the immune response and constitute a source of severe tissue damage.

The implication of miRNAs, small non-coding RNAs of approximatively 20–22 nucleotides, as fine regulators of gene expression in the modulation of neutrophil pro-inflammatory functions is only beginning to emerge. In that context, overexpression of miRNA-451 has been associated with a decrease of neutrophil chemotaxis through the inhibition of p38 MAPK phosphorylation and an attenuation of arthritis severity [18]. To our knowledge, the impact of miRNAs on the regulation of cytokine secretion by neutrophils has not yet been thoroughly investigated despite the importance of miRNAs and their potential impact on cytokine secretion in the pathophysiology of autoimmune diseases. 

In this study, we show that S100A8/A9-P are present in large amounts in synovial fluid from RA patients, emphasizing the fact that this form is probably linked to the pro-inflammatory activities of S100A8/A9 in this pathological context. 

We investigate whether S100A8/A9-P are associated with alterations in the profile of miRNAs, which may regulate cytokine secretion. Our data reveal that miR-146a and miR-155-5p are induced by S100A8/A9-P stimulation in neutrophil-like HL-60 cells through activation of TLR4 signaling pathways. For the first time, we provide evidence for an effect of these two miRNAs in the regulation of S100A8/A9-P-induced cytokine secretion. Overexpression of miR-146a-5p and miR-155-5p using miRNA mimics results in a decrease of cytokine secretion underlining the potential anti-inflammatory role of these two miRNAs when upregulated.

Our study provides a novel insight in the importance of miRNAs in the regulation of crucial pro-inflammatory neutrophil functions and shows their importance in ensuring the fine balance in the inflammatory response in which neutrophils are involved. 

## 2. Results

### 2.1. S100A9 Is Phosphorylated in Synovial Fluids from Rheumatoid Arthritis Patients

Recently, it was shown that neutrophil pro-inflammatory functions mediated by S100A8/A9 are tightly dependent on the phosphorylation state of S100A9 [17]. Although this post-translational form of S100A9 could be a determinant player for the development of chronic and autoimmune diseases, the presence of S100A8/A9-P in clinical samples has not yet been investigated to support this assumption. 

Because S100A8/A9 constitute a hallmark of RA and evidence has been provided for a role of S100A8/A9 in RA pathogenesis, we determined whether S100A9-P was also present in the synovial fluids of patients suffering from RA. For that, synovial fluids from RA patients were subjected to western-blot analysis with a polyclonal antibody against phospho-specific S100A9. Synovial fluids from osteoarthritis (OA) patients were used as control since OA is the result of excessive mechanical stress on the joint and not an autoimmune disease like RA.

**Figure 1:**
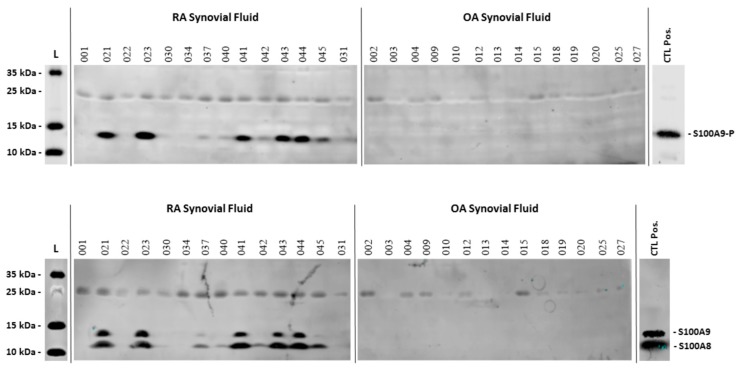
Presence of S100A8/A9-P in synovial fluids from rheumatoid arthritis (RA) and osteoarthritis (OA) patients. 50 µg of proteins from synovial fluids from 14 RA and 14 OA patients were loaded on a 4–15% Tris-Acetate gel for western blot analysis. S100A9-P was detected with the anti-S100A9-P antibody (custom-made), total S100A9 with the B5 antibody and S100A8 using the EPR3554 antibody. (L = ladder and positive control = leukopak purified S100A8/A9-P).

In all synovial fluids from OA patients no S100A9-P was detected. This was also the case for total S100A9 (including S100A9 and S100A8/A9-P) and S100A8 (Figure 1). In contrast, in almost two-thirds of the synovial fluids from RA patients, a phosphorylated form of S100A9 was present (Figure 1). It is important to note that in the synovial fluids in which S100A9-P were present, total S100A9 and S100A8 was also detected.

### 2.2. S100A8/A9-P Induces the Expression of miRNA in Neutrophil-Like Cells

Recent findings showed that S100A8/A9-P is able to induce the secretion of specific cytokines, contrary to S100A8/A9 [17]. We hypothesise that S100A8/A9-P has an impact on cytokine expression and secretion by regulating the post-transcriptional activity of miRNAs. 

**Figure 2:**
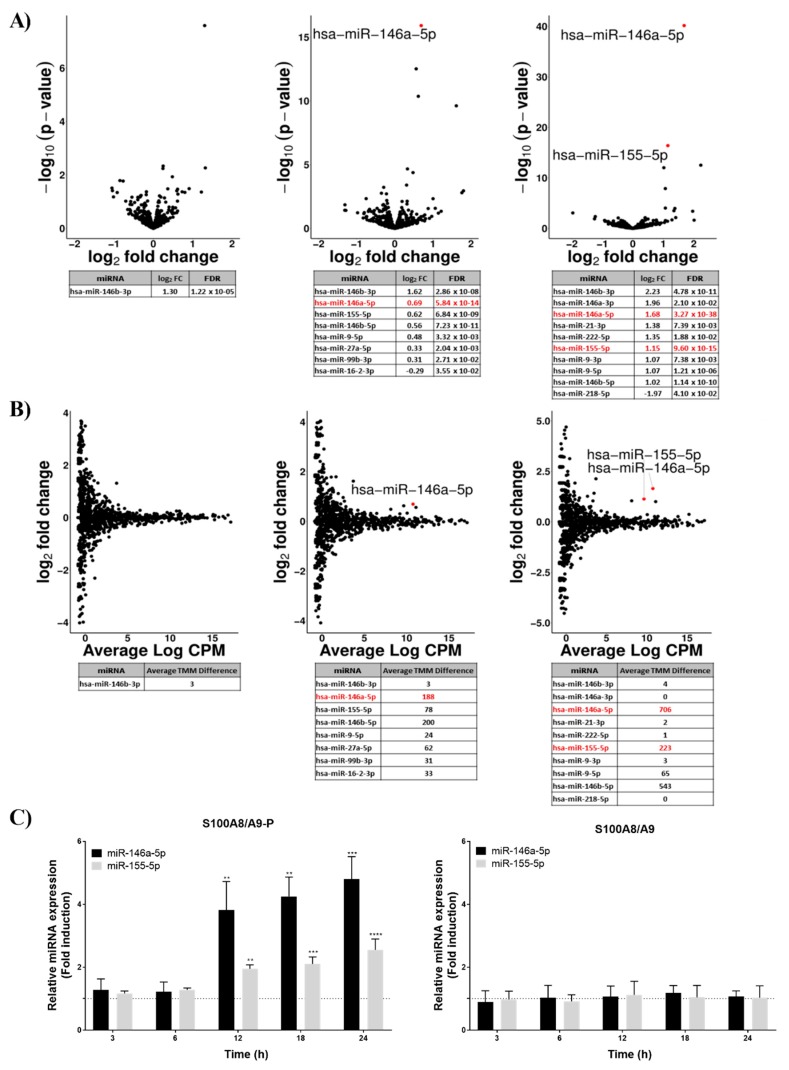
Effect of S100A8/A9-P on miRNA expression in dHL-60 cells. dHL-60 cells were stimulated for 3, 6 and 12 h with 10 µg/mL S100A8/A9-P. (**A**) Volcano plot represents the log2 FC (fold change) (x-axis) against the -log10 p-values (y-axis) for each miRNA identified using miRNA-sequencing. (**B**) MA plot represents the average log CPM (count per million) value (x-axis) against the log2 FC (y-axis) for each miRNA identified using miRNA-sequencing. From three independent experiments, criteria for an effect of S100A8/A9-P on miRNA expression were defined as follows: FDR < 5%, log2 FC ≥ 0.5 and average trimmed mean of M (TMM) value difference between non-stimulated dHL-60 cells and S100A8/A9-P stimulated dHL-60 cells ≥ 100. (**C**) dHL-60 cells were stimulated for 3, 6, 12, 18 and 24 h with 10 µg/mL S100A8/A9-P or S100A8/A9. The expression of miR-146a-5p and miR-155-5p was assessed using qPCR. Data normalization was performed using three reference genes (RNUA1, RNUA5, SCARNA17) and expressed as fold induction compared to the non-stimulated control (dashed line). Results are presented as mean ± SEM of three independent experiments. * *p* < 0.05; ** *p* < 0.01; *** *p* < 0.001; **** *p* < 0.0001.

In a first step, we performed miRNA-sequencing experiments on dHL-60 cells stimulated for 3, 6 and 12 h with 10 µg/mL S100A8/A9-P. Volcano plots (Figure 2A) were created to graphically identify differentially expressed miRNA after stimulation with S100A8/A9-P at different time points in dHL-60 cells. In this aim, log2 fold change (FC) was plotted against the −log10 (*p*-value) and only miRNA with a log2 FC above 0.5 or below −0.5 and a false discovery rate (FDR) below 5% were considered. We found that only one miRNA was overexpressed 3 h after S100A8/A9-P stimulation but the number of miRNAs differentially expressed increased with the time of stimulation (Figure 2A). 

Because volcano plots take into account miRNAs with low-abundance expression, minimal changes in miRNA counts between non-stimulated dHL-60 cells and dHL-60 cells stimulated by S100A8/A9-P can result in an overestimated FC. For this reason, MA plots were also established to graphically visualize the difference between non-stimulated dHL-60 cells and dHL-60 cells stimulated by S100A8/A9-P by including the average count per million (CPM) for both conditions. The MA plots (Figure 2B) showed that certain miRNAs with log2 FC above 0.5 or below 0.5 had a very low average CPM value. miRNAs with an absolute difference of trimmed mean of M-values (TMM, representing the normalized count of detected miRNA) between non-stimulated dHL-60 cells and dHL-60 cells stimulated by S100A8/A9-P above 100, have been identified as miRNAs whose expression was altered by S100A8/A9-P stimulation. In that context, while miR-146a-5p and miR-146b-5p were found to be overexpressed after 6 h of S100A8/A9-P stimulation, miR-155-5p expression was increased only after 12 h of stimulation.

Since their biological importance has been related in the literature to the inflammation process and associated to RA pathogenesis [19,20,21,22,23,24], miRNA-146a-5p and miR-155-5p have been selected as potential important regulators of neutrophil inflammatory functions. The increased expression of miRNA-146a-5p and miR-155-5p observed after RNA-sequencing and bioinformatics analyses required validating. To this end, RT-qPCR was performed from RNAs of dHL-60 cells stimulated by S100A8/A9-P for 3, 6, 12, 18 and 24 h. As expected, expression of miR-146a-5p and miR-155-5p in dHL-60 cells was increased upon S100A9-P stimulation after 12 h of stimulation and continued to increase up to 24 h (Figure 2C). Moreover, it was confirmed that non-phosphorylated S100A9 has no effect on the expression of these two miRNAs. 

### 2.3. S100A8/A9-P Induces miR-146a and miR-155 Expression through TLR4 Signaling Pathways

**Figure 3:**
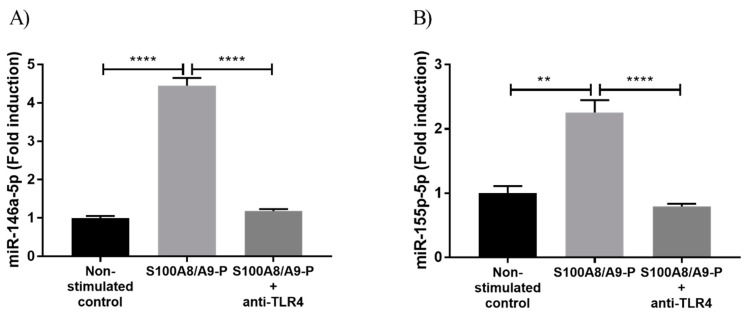
S100A8/A9-P-induced miR-146a and miR-155-5p expression through TLR4 signaling pathway in dHL-60 cells. dHL-60 cells were incubated for 30 min with TLR4 neutralizing antibody, then stimulated with 10 µg/mL S100A8/A9-P for 18 h. 1 µg/mL TLR4 neutralizing antibody was added at 6 h and 12 h of stimulation. Expression of (**A**) miR-146a-5p and (**B**) miR-155-5p were quantified using RT-qPCR. Data normalization was performed using three reference genes (RNUA1, RNUA5, SCARNA17) and expressed as fold induction compared to the non-stimulated control. Results are presented as mean ± SEM of three independent experiments. ** *p* < 0.01; **** *p* < 0.0001.

Since it had been demonstrated that cytokine secretion is mainly regulated through S100A8/A9-P / TLR4 axis [17], we investigated whether the increase of miR-146a-5p and 155-5p expression observed upon S100A8/A9-P stimulation was linked to TLR4 signaling pathway activation. For that purpose, dHL-60 cells were treated with 1 µg/mL TLR4 neutralizing antibodies (or isotopic control, IgG, see Figure S1) for 30 min, then stimulated with 10 µg/mL S100A8/A9-P for 18 h. Then, 1 µg/mL TLR4 neutralizing antibodies or isotypic control were added during the time of stimulation at 6 h and 12 h in order to prevent the loss of TLR4 receptor blocking over time, which could result from antibody degradation or internalization. miR-146a and miR-155 expression was quantified using RT-qPCR. Blockade of TLR4 triggered a strong reduction of miR-146a-5p and 155-5p expression in the range of 75–80% after S100A8/A9-P (Figure 3A,B) but did not completely abolish miRNA expression. This could suggest that TLR4 is not fully inhibited and/or other signaling pathways independent of TLR4 are activated upon S100A8/A9-P stimulation. However, we can conclude that S100A8/A9-P effects are primarily associated to TLR4 signaling pathways.

### 2.4. miR-146a-5p and miR-155-5p Regulate Cytokine Secretion in dHL-60 Cells

#### 2.4.1. Stable Expression of miRNA Mimics

**Figure 4:**
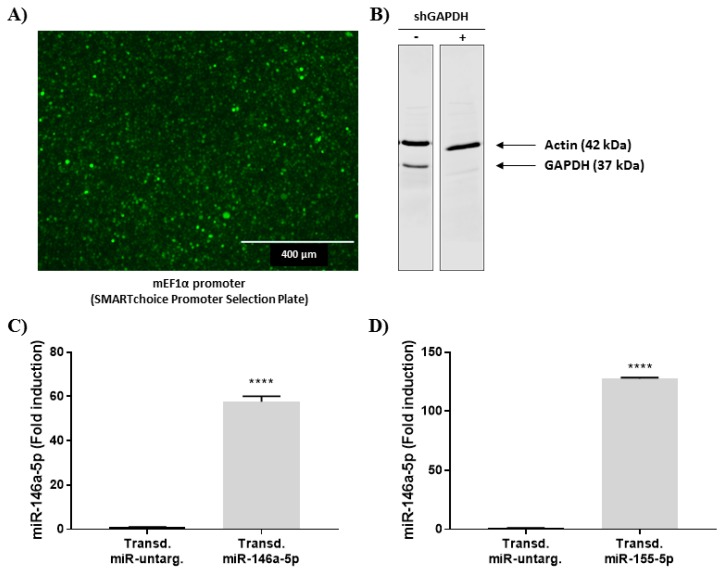
Stable overexpression of miR-146a and miR-155-5p by viral transduction in dHL-60 cells. (**A**) GFP signal is visualized in dHL-60 cells transduced with the GFP vector using the mouse EF1α promoter (MOI of 100) with the SMARTchoice Promoter Selection Plate from Dharmacon. (**B**) Efficiency of GAPDH knockdown in HL-60 cells, transduced with the shRNA directed against GAPDH was analyzed using western blot (10% Tris-Tricine gel). Western blot results are representative of three independent experiments. (**C**,**D**) Quantification of miR146a-5p and miR-155-5p in dHL-60 transduced with both miRNA mimic vectors using the mEF1 promoter. Data normalization was performed using three reference genes (RNUA1, RNUA5, SCARNA17) and expressed as fold induction compared to the respective transduced negative control. Results are presented as mean ± SEM of six (for miR-146a-5) and three (for miR-155-5p) independent experiments. ****p < 0.0001.

To determine the impact of an up-regulation of miR-146a-5p and miR-155-5p on cytokine secretion, stable overexpression was performed through the transduction of viral vectors containing mimics for miR-146a-5p and miR-155-5p, as well as a non-silencing negative control. First, we identified the more effective promoter able to induce the expression of the different vectors using the SMARTchoice Promoter Selection Plate from Dharmacon. With a multiplicity of infection (MOI) of 100, the mouse EF1 promoter (mEF1) was the most appropriate promoter regarding GFP expression and signal intensity (Figure 4A). Therefore, different vectors were designed with mEF1 and transduced into HL-60 cells with a MOI of 100. The transduction efficiency was evaluated by the knockdown of the GAPDH protein, which reached 80%, using a vector containing a shRNA directed against GAPDH (Figure 4B). Finally, overexpression of miR146a-5p and miR-155-5p was validated using RT-qPCR in dHL-60 cells and compared to the non-silencing negative control; the expression of both miRNAs were strongly increased (Figure 4C,D). Taken together, these results clearly show that miR146a-5p and miR-155-5p were successfully integrated into dHL-60 cells.

#### 2.4.2. Effect of miR146a-5p and miR-155-5p Mimics on S100A8/A9-P-Induced Cytokine Secretion

As previously mentioned, S100A8/A9-P can only be obtained in limited quantities. In order to preserve the stock available for the next experiments, we validated the functionality of transduced dHL-60 by stimulating them for 6 h with 100 ng/mL of LPS, which is well-known to mediate its effects through TLR4 signaling pathways, as we observed for S100A8/A9-P, and to quantify cytokine secretion, TNF-α, IL-6, CCL2, CCL3, CXCL8, IL-12b and CCL4 were secreted by cells upon LPS stimulation. Interestingly, the overexpression of miR146a-5p and miR-155-5p mimics greatly reduced the expression of these cytokines compared to the negative control (Figure S2). Moreover, the putative mRNA targets were determined according to Targetscan database predictions and literature with a special focus on TLR4 signaling pathways [21,25]. The identified targets are TLR4, IRAK (1, 2 and 4), NF-kB1, TAB2 and MyD88. Interestingly, overexpression of miR-146a-5p resulted in a reduction of all the LPS-induced targets tested. In contrast, overexpression of miR-155-5p triggered only a decrease in the LPS-induced expression of NF-κB1 and IRAK2 and a reduction tendency for LPS-induced TAB2 and Myd88. These results clearly confirm that miR-146a-5p and miR-155-5p are involved in the regulation of TLR4 signaling mediated by LPS in neutrophils by down-regulating the expression of key actors (Figure S3).

**Figure 5:**
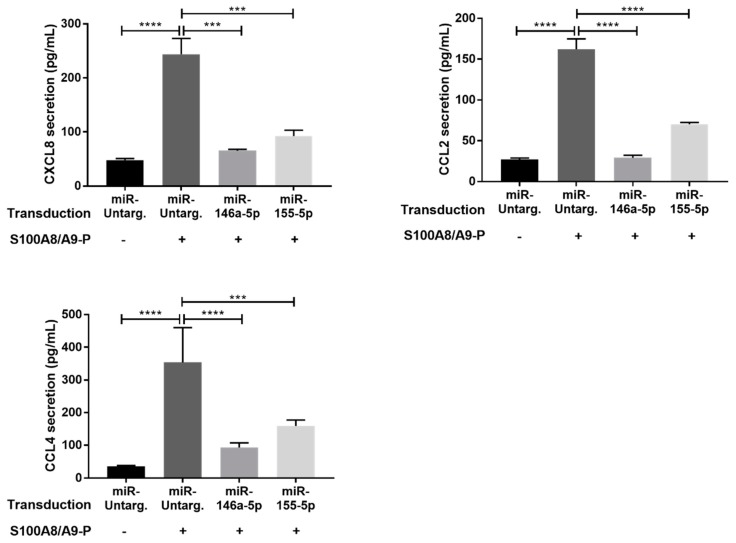
Effect of miR-146a-5p and miR-155-5p mimics on cytokine secretion induced by S100A8/A9-P in dHL-60 cells. Transduced dHL-60 cells with miR-146a-5p and miR-155-5p were stimulated with 10 µg/mL S100A8/A9-P for 6 h. Secretion of CCL2 and CXCL8 was quantified using cytometric bead array and secretion of CCL4 by ELISA. Cell viability for each condition was measured using LDH quantification. The secretion results are presented as mean ± SEM of six independent experiments. *** *p* < 0.001; **** *p* < 0.0001.

Since transduced dHL-60 cells responded to LPS stimulation and mimics were efficient, dHL-60 cells were this time stimulated with 10 µg/mL S100A8/A9-P. Compared to LPS, only the secretion of CCL2, CXCL8 and CCL4 was detected upon S100A8/A9-P cell stimulation (Figure 5). Secretion of each of these cytokines was reduced by the overexpression of miR-146a-5p or miR-155-5p with a more pronounced effect for miR-146a-5p. We can conclude from our results that miR-146a-5p and miR-155-5p regulate S100A8/A9-P-mediated cytokine secretion.

## 3. Discussion

Neutrophils secrete a broad spectrum of pro-inflammatory mediators in the extracellular environment allowing them to exert a pivotal role by modulating immunity and the inflammatory response. In that context, neutrophils are able to release S100A8/A9, which are considered as DAMPs and found in large amounts in biological samples from patients with metabolic inflammatory diseases (e.g., gout) and autoimmune diseases (e.g., rheumatoid arthritis, RA) [26,27,28,29]. Leukocyte recruitment and induction of pro-inflammatory cytokine production are two major functions of DAMPs and represent a feature of disease progression in which S100A8/A9 are involved by inducing inflammation. In this sense, S100A8 and S100A9 have been described to induce cytokine secretion in phagocytes via TLR4 signaling [30,31,32]. However, upon inflammatory conditions, in human neutrophils, S100A9 has been reported to potentiate cytokine release rather than to directly stimulate their secretion [33]. In this way, S100A9, but not S100A8, was found to act as a priming agent by amplifying CXCL8 secretion induced by fMLF or GM-CSF. However, it is important to note that in this study, the effect of S100A8/A9 was not tested and recombinant S100A8 and S100A9 proteins were used, which were devoid of post-translational modifications. Indeed, there is no doubt that the involvement of S100A8 and S100A9 in the regulation of inflammatory functions depends, among other things, on the monomeric and oligomeric forms of S100A8 and S100A9 [34] as well as their post-translational modifications [35,36]. In support of this affirmation, it has been proposed that oxygen derivatives induce post-translational modifications of S100A8/A9, through which the protein complex can scavenge released ROS and thus limit the propagation of inflammation [37,38].

Besides the potential anti-inflammatory role of S100A8/A9, attributed to its oxidation state, the pro-inflammatory effect of S100A8/A9 could be dependent on the phosphorylated state of S100A9. Indeed, it has been recently described that S100A8/A9-P is able to induce cytokine secretion in dHL-60 cells in a TLR4-dependent manner [17]. However, how S100A8/A9-P can regulate such a determinant function remains unknown and has never been investigated despite the importance that this form of the complex could have in the pathogenesis of chronic inflammatory diseases. To reinforce this assumption, our data provides evidence that S100A8/A9-P are found in large amounts in synovial fluids from RA patients and are probably able to trigger the release of cytokines using neutrophils present in the synovial fluid, thus amplifying the inflammatory state.

It is important to note that the phosphorylated form of S100A9 was present in two thirds of synovial fluids from RA patients as well as total S100A9 and S100A8. Determination of the ratio between phosphorylated and non-phosphorylated S100A8/A9 in those synovial fluids could potentially be used as an additional predictive marker for a more precise assessment of disease progression. Indeed, S100A8/A9 has been demonstrated to actively participate in RA pathogenesis [10,11,17,30]. Based on our previous results [17], we claim that phosphorylated S100A9 bears the pro-inflammatory potential of S100A8/A9. In that context, the presence or absence of phosphorylated S100A9 in synovial fluids from RA patients could be directly linked to the degree of RA progression. Indeed, the absence of S100A8/A9-P in the samples could correspond to an early stage of the disease, while the presence of S100A8/A9-P could mean that the disease is in a more advanced stage. To confirm or inform this hypothesis, the amount of S100A8/A9-P needs to be attributed to the rheumatoid factor (for example) and more samples need to be collected (at least 100) to be subjected to analysis. Moreover, all the samples originated from treated patients and consequently the presence of S100A8/A9-P could be dependent on the patients’ responsiveness to the therapy.

In the past few years, emphasis has been put on the study of miRNAs in the regulation of multiple biological processes. Large advances have been made on the relationships between aberrant miRNA expression and carcinogenesis, but their role in the development of autoimmune diseases remains poorly understood. Because neutrophils are key cells associated with such types of diseases, an increase of knowledge concerning the regulation of pro-inflammatory functions of neutrophils by miRNA may be beneficial to define new strategies of therapy. Until now, the most evident impact of miRNA in the regulation of neutrophil functions has been shown on the chemotaxis and concerned miRNA-451 [18].

Here, we show that miRNA-146a and -155-5p are dysregulated upon S100A8/A9-P stimulation and control cytokine secretion via TLR4 signaling pathways in a neutrophil-like model. The decrease or stabilization of cytokine expression over time coincides with an increase of miR-146-5p and miR-155-5p expression (Figure S4). These data support the assumption that these two miRNAs seem to be involved in the negative feedback loop of cytokine expression.

Unfortunately, gene studies in primary neutrophils remain challenging, since they are always non-genetically modifiable due to their status of terminally differentiated cells. An alternative consists of using neutrophils from knock-out mice to study the mechanisms underlying neutrophil functions, but we need to keep in mind that the profile of cytokine secretion in mice is different from that in humans [39]. Moreover, in mice S100A9 is not phosphorylated due to a non-conserved Thr113 residue [40]. For these reasons, we decided to use the dHL-60 cell-like neutrophil model since these cells have been used successfully to characterize the effect of gene/miRNA upregulation or knockdown and it has been reported that neutrophils and dHL-60 cells show a similar profile of cytokine secretion upon LPS stimulation [41]. Overexpression of miRNAs by lipofectamine-based mimic transfection was ineffective in dHL-60 cells (data not shown). This could be explained by a defective incorporation of the mimics into the RISC complex and thus the absence of repression of target mRNA. To overcome this problem, stable overexpression of miR-146a-5p and miR-155-5p through viral transduction has been realized. Moreover, overexpression of miR-146a-5p and miR-155-5p triggered a strong reduction of LPS- and S100A8/A9-P-mediated CCL2, CCL4 and CXCL8 secretion.

Dysregulation of miR-146a-5p and miR-155-5p miRNAs have been shown to be linked to RA pathogenesis and predominantly elevated in RA patients and arthritis models [19,20,21,22,23,24]. Moreover, it has been pointed out that these two miRNAs can participate to the regulation of MyD88-dependent TLR signaling and cytokine secretion in peripheral blood mononuclear cells and monocytes [42,43].

It is currently not clear whether miRNAs are promoting or inhibiting the RA progression [44]. In macrophages from RA patients, miR-155 was shown to be upregulated and to promote the release of IL-6 trough SHIP-1 inhibition [24].

According to our data, S100A8/A9-P, which is present in synovial fluids from RA patients, lead to an increase of miR-146a and miR-155-5p through TLR4 signaling pathways. An overexpression of these two miRNAs results in a sustained decrease of cytokine secretion induced by S100A8/A9-P. Therefore, the control of miR-146a and miR-155-5p expression levels in neutrophils may offer a promising anti-inflammatory option to reduce excessive inflammation in the context of chronic inflammatory diseases.

## 4. Materials and Methods

### 4.1. Reagents and Antibodies

RPMI-1640, L-glutamine, penicillin and streptomycin were obtained from Life Technologies (Gent, Belgium). Fetal bovine serum was purchased from Sigma-Aldrich (Bornem, Belgium). A BCA Protein Assay kit was purchased from ThermoFischer Scientific, (Erembodegem, Belgium). If not specifically mentioned, all the experiments were performed using physiological salt solution (PSS) with the following composition: NaCl 115 mM, KCl 5 mM, KH_2_PO_4_ 1 mM, D-Glucose 10 mM, MgSO_4_ 1 mM, CaCl_2_ 1.25 mM and HEPES-Na 25 mM, pH 7.4. Chemicals were of analytical grade and obtained from Merck (Darmstadt, Germany).

### 4.2. Cell Culture

The human promyelocytic leukemia HL-60 cell line (ATTC, #CCL-240) was cultured in Roswell Park Memorial Institute (RPMI) 1640 medium supplemented with 2 mM L-glutamine, 10% complement heat-inactivated fetal bovine serum (FBS), 100 μg/mL streptomycin and 100 U/mL penicillin. Cells were incubated at 37 °C, 5% CO_2_ and passaged every 3 days. HL-60 cells were differentiated into dHL-60 by the addition of 1.3% *v*/*v* dimethylsulfoxide (DMSO) for 4.5 days [45].

### 4.3. Collection of Synovial Fluids

Synovial fluids from RA and osteoarthritis (OA) patients were collected in collaboration with LIMIDRA (Luxembourg Immune-Mediated Inflammatory Disease Research Association) and CHU (Centre Hospitalier Universitaire) of Brest, France.

Synovial fluids from RA and OA patients were collected under written informed consent from all participants, following the good clinical and ethical practices approved by the Ethics Review Panel (ERP 17-005 NEUTRA, 14 June 2017) of the University of Luxembourg according to the guidelines of the “Comité National d’Ethique de Recherche” (CNER 201701/07, 28 February 2017) and the “Commission nationale pour la protection des données” (CNPD) from Luxembourg.

Synovial fluids were treated with 20 U/mL hyaluronidase (Carl Roth, Karlsruhe, Germany) for 30 min at 37 °C. Then, synovial fluids were filtered through 100 µm cell-strainers (Greiner Bio-One, Frickenhausen, Germany) centrifuged at 300 *g* for 15 min and treated with SigmaFast protease/phosphatase inhibitor cocktail (Merck, Darmstadt, Germany). The synovial fluids acellularity was checked using microscopy prior to determining the protein concentration using a Pierce 660 protein assay kit (Thermo Fisher Scientific, Erembodegem, Belgium). Finally samples were alicoted and stored at −80 °C.

### 4.4. Western Blot of Synovial Fluids

50 µg of synovial fluid samples were centrifuged and loading buffer (Tris 200 mM, pH 8.8; SDS 2%; Glycerol 10%; DTT 100 mM; β-mercaptoethanol 1%) was added before heating for 10 min at 96 °C. Then samples were run on a Tris-Acetate gradient gel (4‒15% acrylamide). Proteins were then either visualized on gel using Coomassie blue staining or electrotransferred to a PVDF membrane (Merck-Millipore, Overijse, Belgium). For the immunodetection of S100A9, mouse mAb B-5 (Santa Cruz, Heidelberg, Germany) was used, while a custom-made rabbit pAb (Thermo Fisher Scientific, Erembodegem, Belgium) was used to detect the phosphorylated form of S100A9. For the immunodetection of S100A8, rabbit mAb EPR3554 (Abcam, Cambridge, UK) was used. Finally, anti-rabbit or anti-mouse coupled with IRDye 800 CW or IRDye 680 (LI-COR Bioscience, Lincoln, NE, USA) were used as secondary antibodies for fluorescent western blot detection using the Odyssey Infrared Imaging system (LI-COR Biosciences, Lincoln, NE, USA).

### 4.5. Purification of S100A8/A9 and S100A8/A9-P

S100A8/A9 and S100A8/A9-P were purified from human granulocytes according to the method published by van den Bos et al. [46] and already described [17]. Briefly, granulocytes were purified from fifty human leukopaks, pooled together and lysed by sonication. The cytosolic fraction containing the majority of S100A8 and S100A9 proteins were obtained by ultra-centrifugation (200,000 g for 1 h at 4 °C). Total protein concentration of the cytosolic fractions was quantified with PIERCE 660 nm Protein Assay (Thermo Fischer Scientific, Erembodegem, Belgium) and adjusted to 3 mg/mL using buffer A solution (Tris 50 mM, EDTA 1 mM, EGTA 1 mM, dithiothreitol, 1 mM, pH 8.5). Because S100 proteins are soluble in ammonium sulfate solutions, a substantial portion of unwanted proteins were removed by 70% *w*/*v* ammonium sulfate precipitation for 2 h at 4 °C followed by centrifugation at 10,000 g for 30 min at 4 °C. The S100A8/A9 enriched supernatants were retrieved and dialyzed against buffer A overnight at 4 °C with a buffer refreshment of four times. In the final purification step, S100A8/A9 and S100A8/A9-P were purified by FPLC using an anion exchange column (HiTrap Q XL, GE Healthcare Life Sciences, Diegem, Belgium) and eluted with a gradient of NaCl 0–0.4 mM at a flow rate of 1 mL/min. While pure S100A8/A9 was eluted at NaCl concentrations in the range of 0.12‒0.135 mM, S100A8/A9-P was obtained using a NaCl concentration in the range of 0.25‒0.27 mM. Protein purity in each eluted fraction was determined using SDS-PAGE under reducing conditions and visualized using Coomassie blue staining. Additionally, presence of a potential contamination by S100A12 was determined by western blot [47]. Finally, S100A8/A9 or S100A8/A9-P fractions were pooled and concentrated to a workable concentration using Amicon^®^ Ultra 3K Centrifugal Filter Units (Merck, Darmstadt, Germany). To exclude endotoxin contamination in the S100 preparations, a LAL Chromogenic Endotoxin Quantitation test (Pierce LAL Chromogenic Endotoxin Quantitation Kit, Thermo Fisher Scientific, Erembodegem, Belgium) was performed. Since concentrations as low as 0.002 ng/mL and 0.003 ng/mL of LPS were found in the S100A8/A9 and S100A8/A9-P batches respectively, we considered our preparations to be endotoxin-free.

### 4.6. RNA Extraction and Reverse Transcription

Total RNA from dHL-60 cells was extracted using a Quick-RNA Miniprep Kit (Zymo Research, Irvine, CA, USA) and according to the manufacturer’s instructions. Reverse transcription of mature miRNA was performed using 0.5 μg total RNA and the miScript II RT kit (Qiagen, Venlo, The Netherlands) according to the manufacturer’s instructions.

### 4.7. miRNA Sequencing and Analysis

miRNA sequencing was performed using Exiqon (Vedbaek, Denmark). Next-generation sequencing (NGS) libraries were prepared, quantified and sequenced using single-end reads with the Illumina Nextseq 500 instrument. Quality control, alignment and downstream analysis was provided by Exiqon.

### 4.8. Quantitative Real-Time PCR

qPCR was performed using primers for miR-146a-5p (Qiagen, MS00003535) and miR-155-5p (Qiagen, MS00031486) as well as for reference genes RNU1A (Qiagen, MS00013986), RNU5A (Qiagen, MS00013993) and SCARNA17 (Qiagen, MS00014014) with iTaqTM Universal SYBR Green Supermix (Bio-Rad, Hercules, CA, USA) in a QuantStudio 12K Flex real-time PCR machine (Thermo Fischer Scientific, Erembodegem, Belgium) using the following program: 2 min at 95 °C followed by 40 cycles of 3 s at 95 °C and 20 s at 60 °C.

For cytokine RNA expression, the following primers were used: CCL2 (*forward, 5′-*CTGCTCATAGCAGCCACCTT-3′; reverse, 5′-GCTTCTTTGGGACACTTGCT-3′), CCL4 (*forward, 5′-* CACCAATGGGCTCAGAC-3′; reverse, 5′-GCTGCTGGTCTCATAGTAATC-3′), CXCL8 (*forward, 5′-* ACAAGAGCCAGGAAGAAAC-3′; reverse, 5′-AACTGCACCTTCACACAG-3′), Actin (*forward, 5′-* GCCCTGAGGCACTCTTCCA-3′; reverse, 5′-TGTTGGCGTACAGGTCTTTGC-3′), B2M (*forward, 5′-* AAGCAGCATCATGGAGGTTT-3′; reverse, 5′-TGGAGCAACCTGTCAGATA-3′) and Gus (*forward, 5′-* CAAGAGCCAGTTCCTCATCA-3′; reverse, 5′-TTGAAGTCCTTCACCAGCAG-3′). qPCRs were performed using the SYBR^®^ Select Master Mix (Thermo Fischer Scientific, Erembodegem, Belgium) in a QuantStudio 12K Flex real-time PCR machine (Thermo Fischer Scientific, Erembodegem, Belgium). The cycling protocol was as follows: 3 min at 50 °C and 3 min at 95 °C followed by 40 cycles of 3 s at 95 °C and 30 s at 61 °C.

Relative expression of the miRNAs and mRNA of interest were normalized against the three reference genes using the qbase + qPCR analysis software (Biogazelle, Zwijnaarde, Belgium—www.qbaseplus.com).

### 4.9. TLR4 Receptor Neutralization

dHL-60 cells (2 × 10^6^ cells) were cultured in 12-well plates and pretreated with 1 μg/mL anti-TLR4 (R&D Systems, Minneapolis, MN, USA) or 1 μg/mL IgG isotypic control (Jackson ImmunoResearch, Sufolk, UK) for 30 min. After pretreatment, dHL-60 cells were stimulated with 10 μg/mL S100A8/A9-P for 18 h. 1 μg/mL anti-TLR4 or IgG isotypic control was added twice (every 6 h) during the stimulation.

### 4.10. Viral Transduction of miRNA Mimics in dHL-60

The most suitable promoter (mEF1α promoter) for HL-60 cells was determined using SMARTchoice Promoter Selection Plate from Dharmacon (Lafayette, CO, USA). The production of viruses was performed using the Lenti-X Packaging Single Shots (VSV-G) transfection kit (Takara-Bio, Saint-Germain-en-Laye, France) plus mEF1α promoter based lentiviral plasmids (Dharmacon, Lafayette, CO, USA) containing the expression sequence for miR-146a-5p or miR-155-5p or shRNA against GAPDH or a non-targeting miRNA. Briefly, HEK 293T cells were seeded at 50,000 cells per cm^2^ in fibronectin-coated Petri dishes. After 8 h, plasmids were added to the premixed viral plasmids/transfection reagent and dispensed on HEK 293T cells. After 48 and 72 h, the virus-containing medium was collected, pooled and concentrated by overlay on a sucrose-containing buffer (10% sucrose *p*/*v*, Tris-HCl 50 mM, pH 7.4, NaCl 100 mM, EDTA 0.5 mM) at a 4:1 v/v ratio and centrifuged at 9500 *g* for 4 h at 4 °C. Hank’s salt solution (Gibco) was added to the semi-dried pellet for re-suspension and placed overnight in the dark at 4 °C [48]. The virus solution was tittered using the Lenti-X GoStix Plus (Takara-Bio, Saint-Germain-en-Laye, France) according to the manufacturer’s recommendations. A concentration of 10^7^ to 10^9^ IFU/mL was generally obtained and virus was directly used or stored at −80 °C in 10^7^ IFU fraction.

For HL-60 infection, 0.1 × 10^6^ cells per well were loaded in 24-well plates pre-coated overnight with a solution of ACD-A (citrate dextrose form A) containing 20 µg/mL RetroNectin. 100 µL virus (~10^7^ IFU, MOI of 100) containing DEAE-dextran (6 µg/mL final concentration) was added to each well, and then the plate was centrifuged at 300 g for 5 min and put back in the incubator at 37 °C and 5% CO_2_ for 6 h [49]. After this incubation time, the media was exchanged and the cells were left for 2 days in an incubator at 37 °C and 5% CO_2_ before starting the selection with 0.3 µg/mL puromycin for 21 days. To estimate transduction efficiency, GFP signals in the cells were visualized using microscopy (Evos, Thermo Fischer Scientific, Erembodegem, Belgium).

### 4.11. Measurement of Cytokine Secretion by Cytometric Bead Array (CBA) and ELISA

1 × 10^6^ cells/mL transduced HL-60 cells were differentiated and stimulated for 6 h with S100A8/A9-P (10 μg/mL). Fresh supernatants were immediately collected for subsequent quantitative measurements of cytokine secretion using Cytometric Bead Array (CBA, BD Biosciences, Erembodegem, Belgium). The multiplex standard curve composed of a mixture of 11 cytokine standards was determined using serial dilutions, according to the manufacturer’s instructions. The mixture of selected capture beads was prepared and added to supernatants for 1 h. The following beads were used: C-C motif chemokine (CCL) 2 (MCP-1, bead D8), CCL3 (MIP-1α, bead B9), CCL5 (RANTES, bead D4), IL-1α (bead D6), IL-1β (bead B4), IL-6 (bead A7), IL-8 (CXCL8, bead A9), IL-12b (bead E5), IFN-γ (bead E7), TNF-α (bead C4) and TNFRII (bead C5). Then, a mixture of phycoerythrin detection reagents was added to each sample. After 2 h of incubation, samples were carefully washed before flow cytometry acquisition on a BD FACSCanto II system (BD Biosciences, Erembodegem, Belgium), adjusted using the calibration bead procedure from the manufacturer. Results were quantified using standard curves and FCAP ArrayTM software (Soft Flow, MinneapolisMN, USA).

CCL4 secretion was quantified by ELISA (R&D systems) according to the manufacturer’s instructions. A LDH test was performed to check cell viability.

### 4.12. Statistical Analysis

The GraphPad Prism 7 software (GraphPad, La Jolla, CA, USA) was used to perform all statistical analyses. For time course experiments based on multiple stimulatory conditions, a two-way ANOVA analysis was first performed followed by a Tukey’s post-hoc test. For two-group comparison, Student’s *t*-test was applied if normal distributions and equal variances were ascertained. Otherwise, a Mann-Whitney test was done. A *p*-value below 5% was considered significant for all the statistical tests and statistical significance was set as follows: * *p* < 0.05; ** *p* < 0.01; *** *p* < 0.001; **** *p* < 0.0001.

## Supplementary Materials

Supplementary materials can be found at https://www.mdpi.com/1422-0067/20/22/5699/s1.

## Abbreviations

RA	Rheumatoid Arthritis
S100A8/A9-P	Phosphorylated form of S100A8/A9
DAMP	Damage-Associated Molecular Patterns molecules
dHL-60	DMSO-differentiated HL-60 cells
TLR4	Toll-Like Receptor
OA	Osteoarthritis
CBA	Cytometric Bead Array
FC	Fold Change
FDR	False Discovery Rate
CPM	Count Per Million
