# Supplementary material for: miRNAs Regulate Cytokine Secretion Induced by Phosphorylated S100A8/A9 in Neutrophils
Source: Int J Mol Sci. 2019 Nov 14;20(22):5699. doi: 10.3390/ijms20225699 (PMC6887701; doi:10.3390/ijms20225699)
Supplement: Supplementary file 1 [file ijms-20-05699-s001.pdf]

Supplementary Materials:

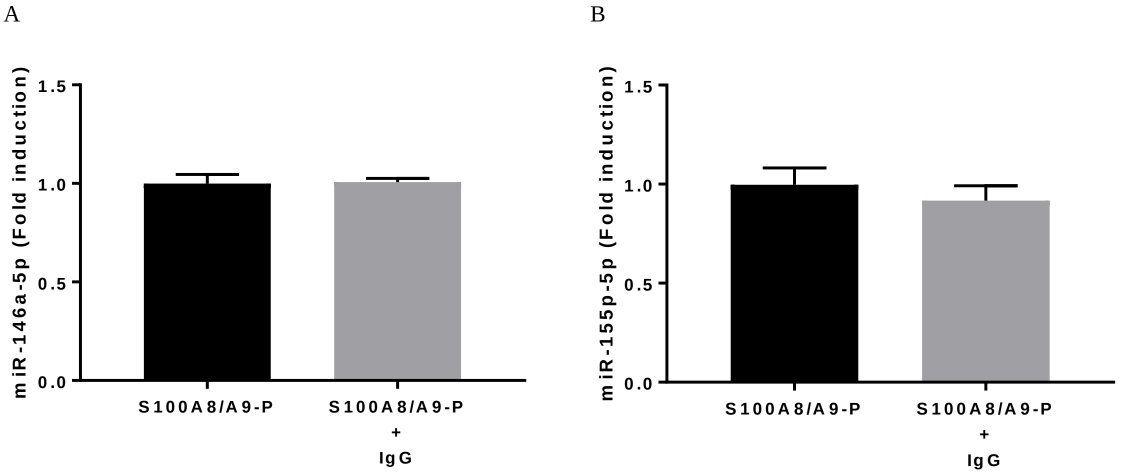

Figure S1. Isotipic control of S100A8/A9-P-induced miR-146a and miR-155-5p expression through TLR4 signaling (Fig 3 of the manuscript). dHL-60 cells were incubated for 30 min with isotopic control (IgG), then stimulated with 10 µg/mL S100A8/A9-P for 18h. 1 µg/mL isotopic control was added at 6h and 12h of stimulation. Expression of **(a)** miR-146a-5p and **(b)** miR-155-5p were quantified by RT-qPCR. Data normalization was performed using three reference genes (RNUA1, RNUA5, SCARNA17) and expressed as fold induction compared to the stimulated control not treated with TLR4 neutralizing antibody. Results are presented as mean ± SEM of three independent experime

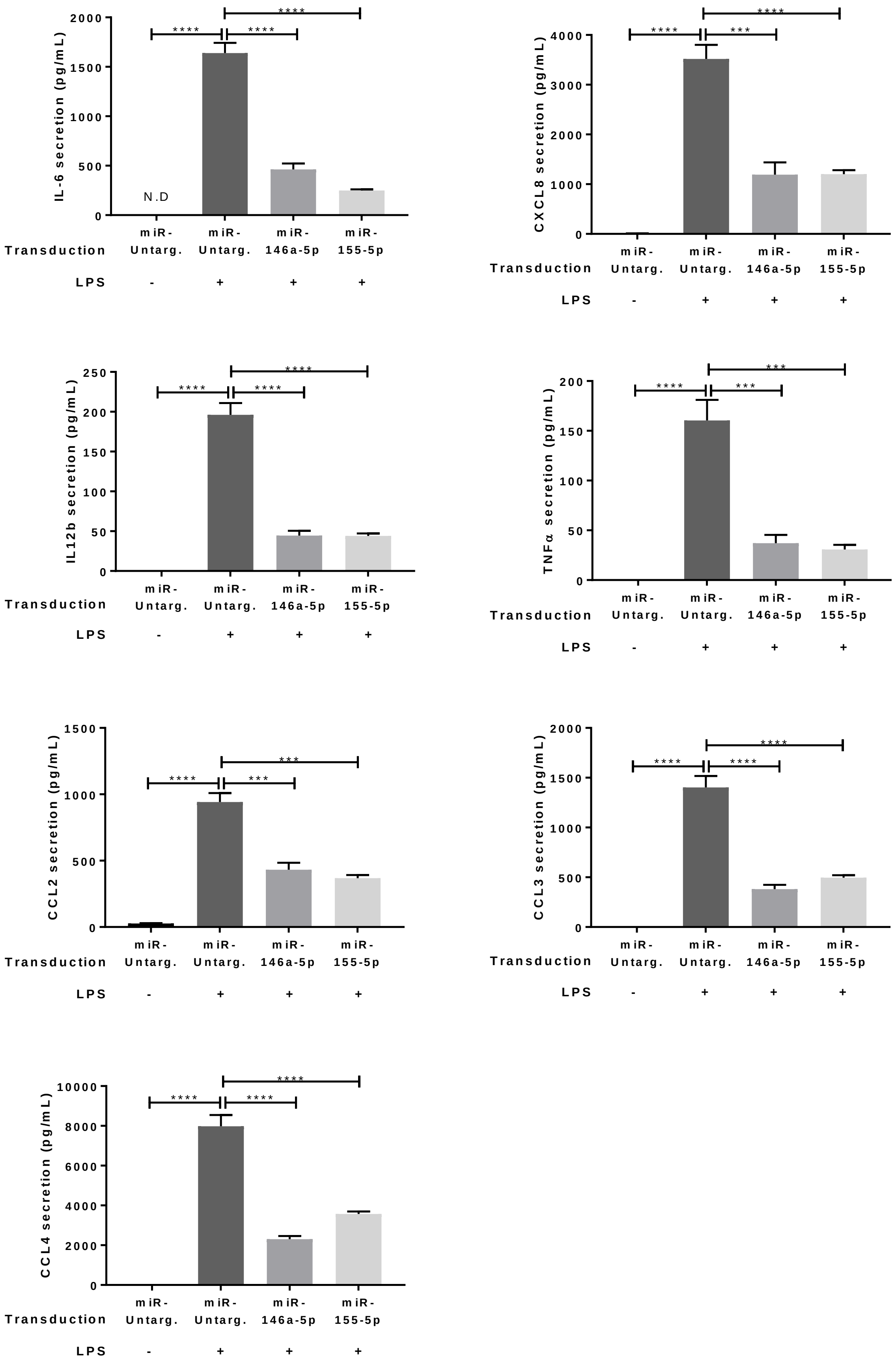

IL-6 secretion (pg/mL)
0
500
1000
1500
2000
N.D
****
****
****
CXCL8 secretion (pg/mL)
0
1000
2000
3000
4000
****
***
****
IL12b secretion (pg/mL)
0
50
100
150
200
250
****
****
****
TNFα secretion (pg/mL)
0
50
100
150
200
****
***
***
CCL2 secretion (pg/mL)
0
500
1000
1500
****
***
***
CCL3 secretion (pg/mL)
0
500
1000
1500
2000
****
****
****
CCL4 secretion (pg/mL)
0
2000
4000
6000
8000
10000
****
****
****
Transduction
miR-Untarg.
miR-Untarg.
miR-146a-5p
miR-155-5p
LPS
-
+
+
+

Figure S2. Effect of miR-146a-5p and miR-155-5p mimics on cytokine secretion induced by LPS in dHL-60 cells. Transduced dHL-60 cells with miR-146a-5p and miR-155-5p were stimulated with 100 ng/mL LPS for 6h. Secretion of TNFα, IL-6, CCL2, CCL3, CXCL8 and IL-12b was quantified by cytometric bead array and secretion of CCL4 by ELISA. Cell viability for each condition was measured by LDH quantification. The secretion results are presented as mean ± SEM of six independent experiments. *$p < 0.05$; **$p < 0.01$; ***$p < 0.001$; ****$p < 0.0001$.

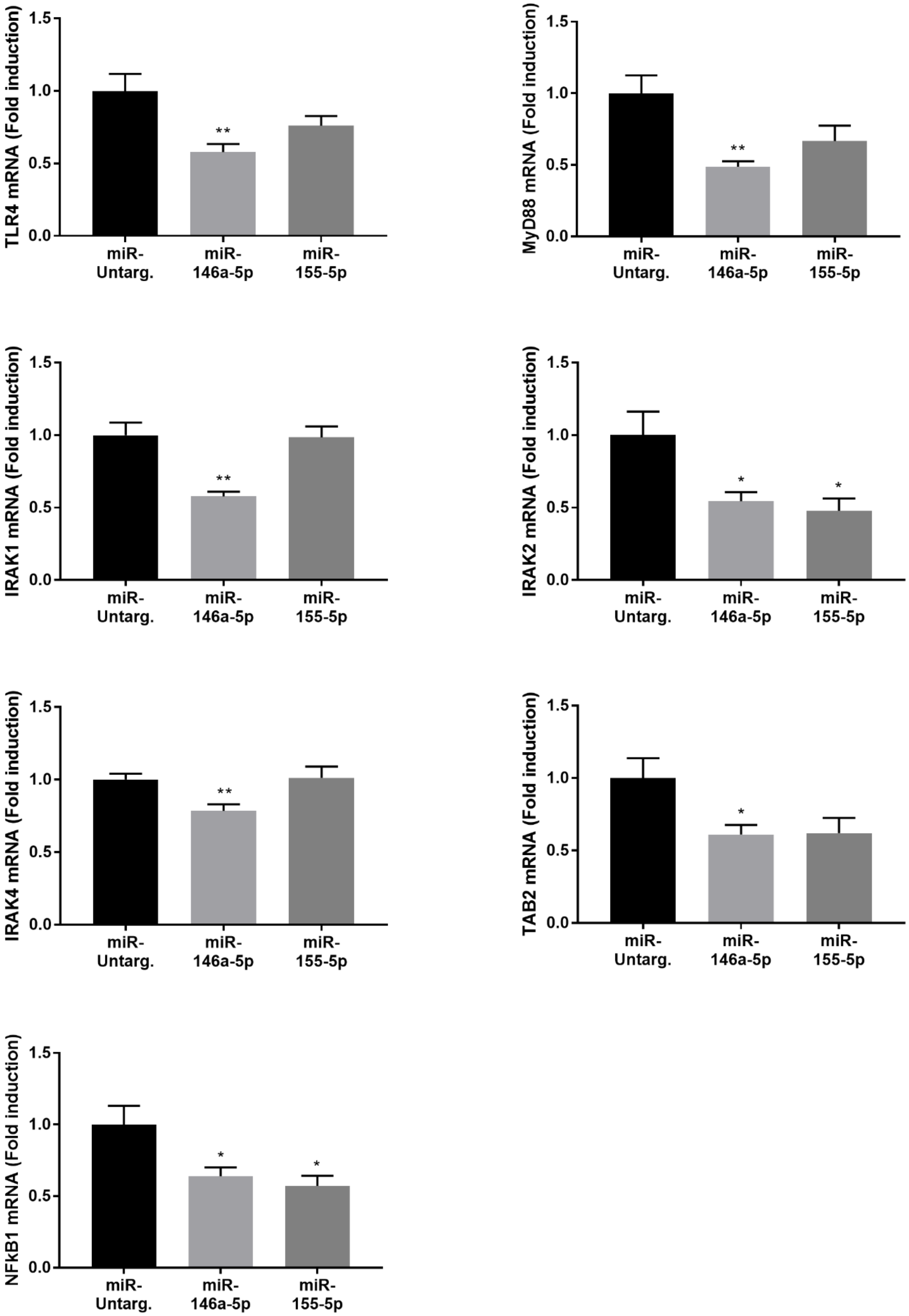

TLR4 mRNA (Fold induction)
MyD88 mRNA (Fold induction)
IRAK1 mRNA (Fold induction)
IRAK2 mRNA (Fold induction)
IRAK4 mRNA (Fold induction)
TAB2 mRNA (Fold induction)
NFkB1 mRNA (Fold induction)
0.0
0.5
1.0
1.5
**
*
miR-
Untarg.
miR-
146a-5p
miR-
155-5p

Figure S3. Effect of transduced miR-146a-5p and miR-155-5p on putative TLR4-dependent mRNA targets. Transduced dHL-60 cells were stimulated for 6 h with 100 ng/mL of LPS. The expression of potential mRNA targets TLR4, MyD88, IRAK1, IRAK2, IRAK4 , TAB2 and NF-kB1 was assessed by real-time PCR. Data normalization was performed using Actin-β, B2M, and Gusβ and expressed as fold induction compared to the stimulated negative control. Results are presented as mean ± SEM of six independent experiments. *$p < 0.05$; **$p < 0.01$; ***$p < 0.001$; ****$p < 0.0001$.

A)

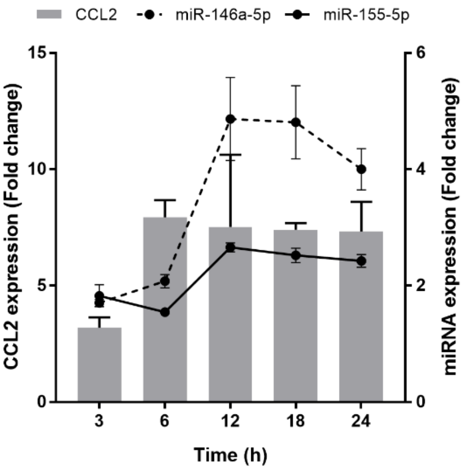

B)

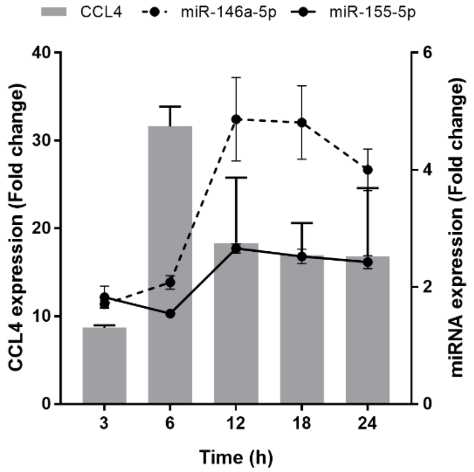

C)

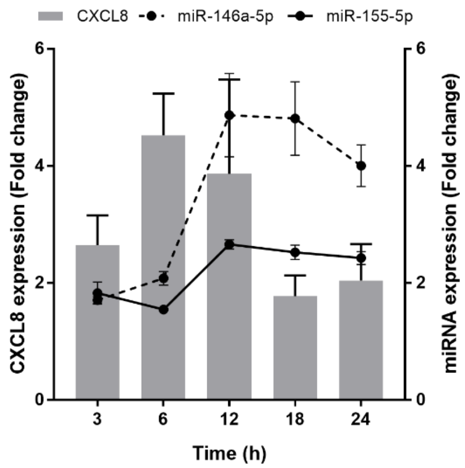

D)

	Statistical analysis				
Time (h)	miR-146a-5p	miR-155-5p	CCL2	CCL4	CXCL8
3	NS	***	NS	NS	NS
6	NS	*	**	****	**
12	****	****	**	*	*
18	****	****	**	*	NS
24	****	****	**	*	NS

Figure S4. Overlay between miRNA expression and cytokine mRNA expression upon S100A8/A9-P stimulation in dHL-60 cells. dHL-60 cells were stimulated for 3, 6, 12, 18 and 24h with 10 µg/mL S100A8/A9-P. The expression of miR-146a-5p and miR-155-5p as well as CCL2, CCL4 and CXCL8 was assessed by qPCR. Data normalization was performed using three reference genes (RNUA1, RNUA5, SCARNA17 for miRNAS and Actin-β, B2M and Gusβ for cytokine expression) and expressed as fold induction compared to the respective non-stimulated control. The results of miRNA and cytokine expression have been superposed for CCL2 (A), CCL4 (B) and CXCL8 (C). Results are presented as mean ± SEM of 3 independent experiments. For more convenience, all statistical analysis were summarized in the table D). * = $p < 0.05$; ** = $p < 0.01$; *** = $p < 0.001$; **** = $p < 0.0001$.
